# Fruit syndromes in *Viburnum*: correlated evolution of color, nutritional content, and morphology in bird-dispersed fleshy fruits

**DOI:** 10.1186/s12862-019-1546-5

**Published:** 2020-01-13

**Authors:** Miranda A. Sinnott-Armstrong, Chong Lee, Wendy L. Clement, Michael J. Donoghue

**Affiliations:** 1 Department of Ecology and Evolutionary Biology and Peabody Museum of Natural History, Yale University, New Haven, CT 06520 USA; 2 Department of Ecology & Evolutionary Biology, University of Colorado—Boulder, Boulder, CO 80309 USA; 3 Department of Fisheries, Animal and Veterinary Sciences, University of Rhode Island, Kingston, RI 02881 USA; 4 Department of Biology, The College of New Jersey, Ewing, NJ 08628 USA

**Keywords:** Fruit color, Seed dispersal, Plant-animal interactions, Fruit syndromes, Temperate forests, Correlated evolution, Trait evolution

## Abstract

**Premise:**

A key question in plant dispersal via animal vectors is where and why fruit colors vary between species and how color relates to other fruit traits. To better understand the factors shaping the evolution of fruit color diversity, we tested for the existence of syndromes of traits (color, morphology, and nutrition) in the fruits of *Viburnum*. We placed these results in a larger phylogenetic context and reconstructed ancestral states to assess how *Viburnum* fruit traits have evolved across the clade.

**Results:**

We find that blue *Viburnum* fruits are not very juicy, and have high lipid content and large, round endocarps surrounded by a small quantity of pulp. Red fruits display the opposite suite of traits: they are very juicy with low lipid content and smaller, flatter endocarps. The ancestral *Viburnum* fruit may have gone through a sequence of color changes before maturation (green to yellow to red to black), though our reconstructions are equivocal. In one major clade of *Viburnum* (Nectarotinus), fruits mature synchronously with reduced intermediate color stages. Most transitions between fruit colors occurred in this synchronously fruiting clade.

**Conclusions:**

It is widely accepted that fruit trait diversity has primarily been driven by the differing perceptual abilities of bird versus mammal frugivores. Yet within a clade of largely bird-dispersed fruits, we find clear correlations between color, morphology, and nutrition. These correlations are likely driven by a shift from sequential to synchronous development, followed by diversification in color, nutrition, and morphology. A deeper understanding of fruit evolution within clades will elucidate the degree to which such syndromes structure extant fruit diversity.

## Background

Fleshy fruits play an essential role in the lives of many plant species, attracting animal dispersers who consume the fruit and carry the seeds away from the parent [1]. This interaction benefits both the plant and animal partners. Animal dispersers receive nutrients and calories by consuming the fleshy pulp of the fruit [2, 3], while plants receive dispersal services, enabling gene flow, escape from predation and pathogens, and range expansion [4, 5]. To communicate that their fruits are ripe and ready to be consumed, animal-dispersed plants have evolved visual displays that include the color of individual ripe fruits, clusters of multiple fruits in an infructescence, and/or secondary structures such as immature fruits or pedicels [1, 6]. Although the colors of fruits are certainly important, color is not the only trait that assists plants in signaling to animals the presence of ripe fruit: odor, fruit size, and the size of clusters of fruits (infructescences) are all also important (e.g., [6, 7]).

The primary hypothesis to explain variation in these visual traits has been selection by animals via their perceptual abilities, behavior, and physiology. According to this hypothesis, most fruits can be classified as either “bird fruits” (small, brightly colored) or “mammal fruits” (large, dull in color, possibly with a husk or rind) [6, 8, 9]. However, this “disperser syndrome hypothesis” is controversial, in part because evidence for selection by dispersers is weak [6, 10–12], and because there are strong associations between fruit color and fruit size that may be attributable to the metabolic costs of producing a large fruit rather than selection by animals [6, 13, 14]. Furthermore, birds and mammals are obviously heterogeneous groups with varying visual systems, body sizes, and abilities to manipulate their food, and the simplistic classification into “bird” or “mammal” syndromes does not adequately reflect fruit trait diversity.

An important question when assessing the extent to which putative fruit syndromes relate to animal dispersers is whether aspects of the visual display communicate anything to animals about a fruit’s nutritional content. For example, do juicy red fruits reliably provide the same nutritional reward across species? The color of a fruit can honestly signal its nutritional content [15–18], or can be deceptive by mimicking a more nutritious fruit [19, 20]. Although there are compelling reasons to expect that animal dispersers select for honest signals [18], this question has seldom been investigated. In the Atlantic rainforest of Brazil, darker fruit color was correlated with a more carbohydrate-rich pulp [18]. In the Mediterranean, however, darker colors were correlated with a lipid-rich reward [13, 17, 21]. These conflicting results suggest that, to the extent that color and nutritional content are correlated, the nature of those correlations may not be universal but particular to individual communities and/or plant clades.

Given confusion about the degree to which fruits fall into the classical bird and mammal syndromes, as well as our limited knowledge of how fruit colors relate to nutritional content, we sought to address fruit function and evolution at a different scale and from a different angle. Instead of asking whether fruits exhibit syndromes of traits according to their dispersers, we simply ask whether fruits within an individual clade exhibit distinct syndromes of any type. Furthermore, we ask how fruit trait diversity — in particular fruit color diversity — has evolved and whether we are able to identify any mechanisms that may underlie fruit color diversification. In this study, we use the flowering plant clade *Viburnum* as a model system to address these questions. *Viburnum* is a group of 163 species of mostly temperate forest shrubs and small trees of the Northern Hemisphere, although some species do occur in tropical regions of the Indo-Pacific as well as in cloud forests of Central and South America [22–25]. All *Viburnum* species produce fleshy, animal-dispersed drupe fruits and are largely bird-dispersed. *Viburnum* fruits are borne in clusters (infructescences) of ~ 20–100 fruits and display a wide variety of mature fruit colors, developmental patterns, and nutritional contents. Excellent knowledge of both the *Viburnum* phylogeny based on chloroplast and nuclear DNA sequences [22, 26, 27] and fruit traits of many species [24, 26, 28, 29] make *Viburnum* ideal for a study of the evolution of fruit syndromes within putatively bird-dispersed species.

**Fig. 1:**
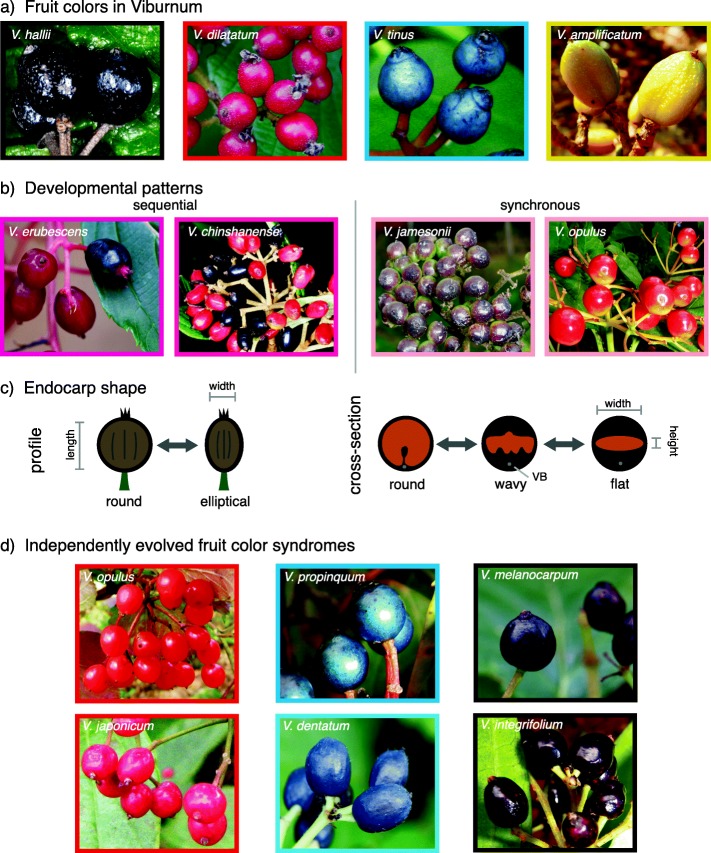
*Viburnum* fruits exhibit a wide variety of fruit colors, developmental patterns, and endocarp shapes. **a** *Viburnum* displays four distinct colors at maturity: black, yellow, red, and blue. **b** Each species also exhibits one of two developmental patterns, either sequential (where immature fruits and mature fruits persist on the same infructescence, and the immature fruits provide a contrasting color to the mature fruits) or synchronous (where all fruits develop at the same time from green to their mature fruit color). **c** Endocarp shape varies in two dimensions: in profile, the endocarp may appear round or elliptical. In cross-section, the endocarp may appear round, flat, or wavy. Endocarp shape was measured according to the labels here (length, width, and height). **d** *Viburnum* fruit colors have evolved independently multiple times. Here, we illustrate these independent origins with example species from each origin. Black-sequential fruits only evolved once, but black-synchronous fruits evolved three times from red-fruited ancestors; shown here are two of those three origins

Mature fruiting displays can be decomposed into three main features in *Viburnum*: the color of mature fruits, the developmental pattern of those fruits, and the presence or absence of immature color stages. The majority of species display black or red fruits at maturity, but some species produce yellow, orange-red, or blue fruits (Fig. 1a). Developmental pattern refers to whether fruits mature synchronously or asynchronously, and this is closely related to whether fruits display immature fruit colors following the green stage (i.e., yellow and/or red). *Sequentially* developing fruits mature one at a time, such that mature fruits and contrasting immature fruits are present in the same infructescence (Fig. 1b). *Synchronously* developing fruits mature more or less at the same time in an infructescence, such that there is not a prolonged period during which there is a distinct color contrast between immature and mature fruits. Most synchronously developing *Viburnum* species develop directly from green to their mature color, though some do go through brief immature color phases [30, 31].

In addition to mature fruit color and developmental pattern, a fruit’s morphological traits and nutritional content form important components of the fruit syndrome. All *Viburnum* species have drupe fruits with fleshy pulp surrounding a single seed that is encased in a hardened inner wall of the ovary, or endocarp [29]. The endocarp functions like a seed in that when a fruit is eaten, the whole endocarp passes through the gut of the animal and the pulp is removed. Endocarps in *Viburnum* range in shape from relatively flat to round, and they vary extensively in the degree to which they are grooved and therefore appear “wavy” in cross section (Fig. 1c) [29, 32]. The nutritional content of the fruits of most *Viburnum* species is unknown, but the few that have been studied have lipid contents ranging from very low (~ 2% dry weight, e.g., *V. opulus*) to very high (> 40% dry weight, e.g., *V. dentatum*) [13, 33–37]. Of the species that have been studied, the vast majority are dispersed by birds although some dispersal by foxes, mustelids, and monkeys has been reported [38–42].

In this paper we address three questions about *Viburnum* fruit evolution in the context of the *Viburnum* phylogeny. First, we examine a set of 29 species in order to assess whether there are syndromes of traits in *Viburnum* fruits that have evolved in a correlated fashion, focusing on fruit color, fruit and endocarp size, and nutritional content. We then scale out to examine the evolution of selected fruit traits across a much larger set of *Viburnum* species. Specifically, we examine correlations in fruit color and endocarp shape across 115 *Viburnum* species to assess their correspondence with our smaller subset of species. Finally, we infer ancestral fruit colors across all of *Viburnum* to trace the evolutionary assembly of fruit syndromes, specifically to test whether paedomorphic evolution could underlie fruit color diversification. Many *Viburnum* species exhibit a marked developmental color sequence, from green to yellow to red to black. In addition to species that exhibit this complete sequence, these colors also characterize the mature fruits of other species (e.g., *V. amplificatum* has yellow fruits, members of the Succotinus clade have red fruits, and members of the Oreinotinus clade have black fruits that for the most part lack intermediate yellow or red stages). Truncation of development in various ways — loss of intermediate red and yellow stages, or loss of the final black stage, for example — could underlie transitions from a sequentially developing fruit to a wide variety of mature fruit colors. We seek to test whether paedomorphic evolution could underlie trait diversification in *Viburnum*, and possibly in other clades as well.

## Methods

### Fruit traits

We collected mature fruits from 29 *Viburnum* species, spanning all major clades and mature fruit color categories (accession numbers in Additional file 1: Table S1). For each species, we sampled fruits from a single individual (except for *V. cylindricum*, where multiple individuals were available and were needed to provide sufficient fruit material). Fruits were obtained from plants in the Arnold Arboretum (Jamaica Plain, Massachusetts, US), Kew Gardens (Richmond, UK), the Cambridge University Botanic Garden (Cambridge, UK), the Berkeley Botanical Garden (Oakridge, California, US), wild collections in Chiapas and Oaxaca, Mexico, and plantings in New Haven, CT. We measured reflectance and morphological traits (fruit and endocarp dimensions) for 20 fruits per species whenever possible, and separated pulp from endocarps for as many fruits as needed to conduct nutritional analyses (Additional file 1: Table S1).

### Reflectance

In most cases, reflectance was measured on the day of sampling. If measuring reflectance was not possible on the day of collection, we kept the fruits chilled and in air-tight containers to prevent drying until reflectance could be measured. Reflectance spectra of 20 fruits per species (when possible; Additional file 1: Table S1) were measured with an OceanOptics USB2000 spectrometer with deuterium and halogen lamps and a Spectralon white reflectance standard (Ocean Optics, Dunedin, FL). Using the R package *pavo* [43]*,* we smoothed the reflectance curve to 5 nm bands, set negative reflectance values to 0, and smoothed background reflectance artifacts. After this processing, we averaged the reflectance measurements of all of the fruits measured per species to generate a mean reflectance spectrum per species. We then modeled each species in bird visual space using the UV-sensitive bird model in the R package *pavo* [43]. This model estimates the stimulation of UV-, short- (blue), medium- (green), and long-wave (red) sensitive cones based on the reflectance spectrum. Using these models of fruit color, we estimated the volume overlap in tetrahedral color space of each pair of color categories.

### Morphology

For each species, we measured fruit length, width, and height for 20 fruits per species (when possible; Additional file 1: Table S1) using Mitutoyo Absolute Digimatic calipers. Length was measured from the base of the fruit to remnants of the calyx (Fig. 1c). Width and height were established based on the dorso-ventral axis of the endocarp (Fig. 1c). In *Viburnum,* the presence of a vascular bundle (centered on the ventral side and running from the bottom to the top of the ovary/fruit) provides a landmark for the measurement of the width and height (the dorso-ventral axis) of the fruit and the endocarp (Fig. 1c). In cross-section, “flat” endocarps, as we define them, are wide but not very high, whereas “round” endocarps are about as wide as they are high. We manually removed the pulp from 20 fruits, soaked them in water for 48 h and shook them for 5 min to remove any remaining pulp. These endocarps were then dried at room temperature and the length, width, and height were measured. We estimated the volume of both the fruit and the endocarp as an ellipsoid, and the volume of the pulp as the volume of the fruit not taken up by endocarp.

### Nutritional content

Fruits were kept chilled at 4 °C until nutritional content could be analyzed; this was completed within 1 week of fruit collection. We separated the pulp from the endocarp, which is of no nutritional value to the disperser, to generate sufficient material for three measurements per species where possible. Overall, we dissected > 2300 fruits with an average of 81 per species (ranging from 7 fruits for *V. bracteatum* to > 450 fruits for *V. propinquum*). We quantified moisture content, ash, lipids, and protein, following the Association of Official Analytical Chemists (AOAC) methods [44]. For moisture and ash content, we dried ~ 1 g of fresh fruit pulp at 110 °C for 24 h; the moisture content is the weight (water) lost after drying. We then combusted the dried sample at 550° for 6 h; the weight of the remaining material was the ash content. To quantify protein content, we employed the Kjeldahl method using the Kjeltec System 1002 (Tecator, Höganäs, Sweden) and estimated the protein content as 6.25 x N. The lipid content was determined using a simple, rapid solvent extraction method adapted for plant tissues and enabling quantification on a relatively small mass of material [45]. We weighed ~ 0.5–1.0 g of fruit pulp and homogenized this in a blender for 90 s with 20 mL of a 2:1 ratio of chloroform:methanol. After extraction, the sample was filtered and combined with 8 mL of NaCl. This prevents emulsion formation and promotes clear separation of the chloroform layer (containing lipids) from the methanol layer. After separation, we dried 6 mL (4 mL for several samples) of the lipid-containing chloroform layer at ~ 80 °C and weighed the resulting mass of lipids. Carbohydrates were taken as the remainder after lipids, protein, and ash were accounted for. We report here the mass of lipids, protein, ash, and carbohydrates based on the *fresh* pulp because dispersers consume fresh pulp rather than dried pulp; however, we also report dry mass values to provide comparability with related studies.

### Phylogeny

For all *Viburnum-*wide trait evolution analyses, we used the phylogeny presented in Landis et al. [22]. In brief, this phylogeny jointly estimates the phylogeny using a combination of molecular sequence data (restriction-site associated DNA sequencing [RAD-seq] data for 118 species, plus chloroplast and nuclear DNA for 153 species), biogeographic information, biome affinity, and morphological trait data. Divergence times were estimated by including five fossil pollen grains in the analysis. Overall, this joint estimation of the phylogeny yielded a maximum clade credibility tree containing all of the currently recognized extant *Viburnum* species, for a total of 163 taxa plus five fossil taxa. This topology closely agrees with most recent estimations of the *Viburnum* phylogeny (e.g., [24, 26–28]), with one major exception. In previous phylogenetic reconstructions, *V. clemensiae* is placed as sister to the remainder of *Viburnum.* In Landis et al. [22], the position of *V. clemensiae* is equivocal. It is either placed as sister to the rest of *Viburnum* or as sister to one of the two major subclades of *Viburnum* (containing Crenotinus + Valvatotinus + Pseudotinus + Urceolata). This difference has little impact on our understanding of fruit color evolution, with the possible exception of our estimation of the root state.

### Identification of syndromes

To identify potential syndromes of fruit traits, we conducted a phylogenetic principal components analysis (PCA) using the R package *phytools* [46, 47] in order to identify whether different fruit color categories (as perceived by humans) occupied different regions of PC space. To incorporate evolutionary relatedness, we used the phylogeny described above [22] and assumed a Brownian motion correlation structure for each trait. We chose five non-color fruit traits to include in the PC analyses that were likely to influence selection on fruit traits by dispersers: lipid content, moisture content, pulp volume, endocarp flatness (width/height ratio), and fruit width. For nutritional content, we included lipid and moisture content. Since carbohydrates were calculated as the remainder after protein, ash, and lipids, it was inappropriate to include both carbohydrate and lipid content as these were highly correlated. Protein and ash both constituted < 10% of fruit nutritional content in nearly all species, and thus are unlikely to be an important factor in selection on fruit traits. For morphology, we chose endocarp flatness, fruit width, and the volume of pulp. The primary feature of endocarps in *Viburnum* is how flat versus round they are in cross-section, which we characterized by including the flatness dimension in our analyses. Fruit dimension variables were all highly correlated with one another, so we chose to include one variable (width) because that dimension likely determines whether the fruit can fit down a bird’s throat [48]. Finally, the volume of pulp (the ratio of endocarp volume to fruit volume) is crucial because that volume determines the nutritional content of the fruit. We performed linear regressions of phylogenetic independent contrasts [49] as implemented in the R package *ape* [50], accounting for multiple comparisons using a Bonferroni correction. All variables were centered and scaled prior to analysis.

In addition to assessing trait syndromes in a subset of species for which we could gather a wide range of data, we also tested for correlated evolution of endocarp shape and mature fruit color across 115 species of *Viburnum*. Endocarp shape data from this broader sample of *Viburnum* were obtained from Clement et al. [32], and includes measurements for endocarp length, width, and height measured the same way as we measured our smaller subset of species. Endocarps for these species came from dried herbarium specimens with 1–3 samples per species. To assess the impact of fruit color category on the log of endocarp shape (specifically, flatness or the width to height ratio), we performed an analysis of variance (ANOVA) in the R package *phytools* and corrected for phylogeny by representing phylogenetic relatedness with a variance-covariance matrix of the dependent variable (endocarp shape). We additionally ran a non-parametric Kruskal-Wallis test in order to test for unequal sample sizes in endocarp shape across fruit color categories.

### Viburnum-wide color classification

Information on mature *Viburnum* fruit colors and developmental patterns was based on our own field studies in all major centers of *Viburnum* diversity, observations in arboreta and botanical gardens, and published sources [30, 31, 51–56]. Even in a well-studied clade like *Viburnum,* there can be considerable disagreement as to the trait states for some species. The descriptions of fruits in floras and monographs usually rely on herbarium collections, which typically note the color of the fruits at the time of collection and may or may not indicate whether that fruit is mature. As a result, there can be inconsistencies between published accounts and field observations. For example, many species of Solenotinus are described as red-fruited in the *Flora of China*, but our own detailed observations of some species (e.g., *V. subalpinum*) demonstrate that they turn black shortly before dispersal or falling off the plant. We present here the results based on our field-based observations.

Above, we identified three relevant traits of mature fruits: color, developmental pattern, and presence or absence of immature color stages. *Viburnum* fruits that develop synchronously generally do not express immature fruit colors, or, if they do, those phases are quite limited. Thus, these two traits are redundant in this case. The fruits of all sequentially developing species are black at maturity, but there are also black-fruited species that mature synchronously (e.g., in the Porphyrotinus clade, *V. acerifolium, V. melanocarpum, V. integrifolium*). All blue- and red-fruited species also mature synchronously with the possible exception of *V. clemensiae*; few fruits are known from this species but they appear to mature to red following a noticeable yellow phase. The developmental pattern of the sole yellow-fruited species, *V. amplificatum* of Borneo, is poorly known, but for the purposes of this study we have classified it as sequential. Thus, while there are many possible combinations of these three traits (color, developmental pattern, and immature color stages), in *Viburnum* we observe only four major fruit color categories: black-sequential (which we have colored purple in all figures), black-synchronous (colored black), red-synchronous (referred to as and colored red), and blue-synchronous (referred to as and colored blue).

### Transition rate analyses

We estimated the number of transitions as well as the rates of transition between the four major fruit color categories (black-synchronous, black-sequential, red, and blue) using stochastic character mapping [57], as implemented in the R package *phytools* [46]. We simulated 1000 character histories from which we calculated the average number of transitions between states, as well as the rates of transition between each color category.

### Ancestral state reconstruction

In order to test the paedomorphy hypothesis, we inferred ancestral fruit color category using a phylogenetic tree for *Viburnum* [22]. We first implemented a paedomorphy model in maximum likelihood. We developed a transition matrix between each fruit color category that is congruent with the paedomorphy hypothesis (Additional file 1: Tables S2, S3) and then compared this model to the best of three other models of evolution (equal rates, symmetric, and all-rates different). Paedomorphic evolution could occur by truncation of the final color stage (e.g., a black-sequential fruit could lose the final black stage and thereby evolve red, or lose black and red to evolve yellow). Alternatively, intermediate stages could be truncated (e.g., a black-sequential fruit could lose the intermediate red stage and thereby evolve a black-synchronous fruit). Thus, we allow direct transitions from black-sequential to black-synchronous, red, and yellow. However, black-sequential fruits cannot transition directly to blue fruits, which are synchronous and produce their blue color via an alternative mechanism (structural color produced by lipid droplets embedded in the cell wall) [58, 59] and are most likely derived from ancestral black-synchronous fruits. We disallowed all transitions that did not involve a paedomorphic change, although we note that the transition matrix is symmetrical (such that if black-sequential can evolve into red, red can also evolve into black-sequential).

We implemented this model in both maximum likelihood [46] and parsimony (in Mesquite v. 3.3). In maximum likelihood, we defined a transition matrix according to the allowable transitions described above (Additional file 1: Table S2). For parsimony, we used a “step matrix” where the cost of a transition was counted as the sum of changes in fruit color, development pattern, or the addition of a structural blue color that occurred (Additional file 1: Table S3; [58, 59]). For example, the transition from black-sequential to blue fruits included a loss of intermediate color stages, the evolution of synchronicity, and the addition of a blue structural color (for a total cost of three). Likewise, the transition from red to black synchronous includes the addition of a black stage and the loss of a red stage but no change in synchronicity because both fruit types are already synchronous (for a total cost of two).

We then tested our maximum likelihood model against the most commonly used models (equal rates, symmetric, and all-rates-different) and selected the model with the lowest corrected Akaike information criterion (AICc). We compared the resulting paedomorphy model to that best model.

All data and analytical code are publicly available at Data Dryad: 10.5061/dryad.h44j0zpft.

## Results

### Color, nutrition, and morphology

**Fig. 2:**
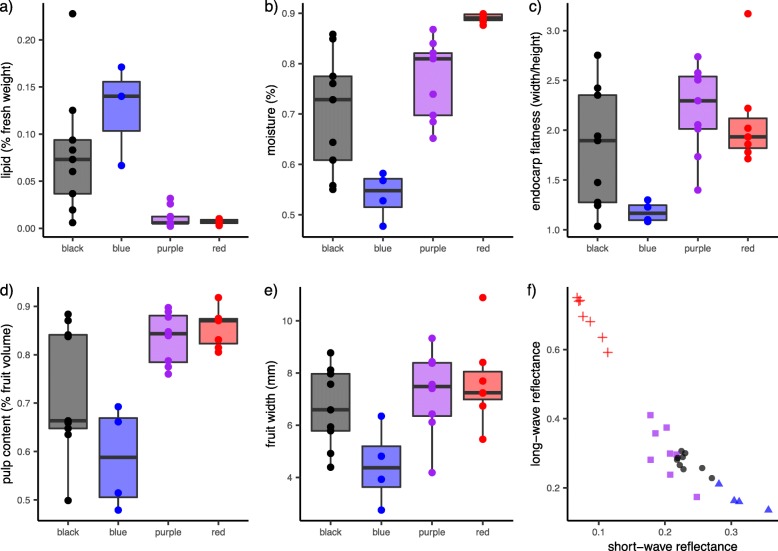
We used five fruit traits for our analyses testing the existence of syndromes in *Viburnum*. The observed species values, differentiated into fruit color category, are displayed here: **a** lipid content, **b** moisture content, **c** pulp volume, **d** endocarp flatness (width/height ratio), and **e** fruit width. Each point indicates the measured value for a species. In **f**, we display the two main variables describing fruit color that are relevant to *Viburnum*: short-wave reflectance (reflectance in the blue region of the spectrum) and long-wave reflectance (reflectance in the red region of the spectrum)

Modeling of fruit color reflectance in UV-sensitive bird visual space yielded differentiation between red-fruited species (which occupied a distinct region of visual space) and the remainder (Additional file 1: Figure S1). A gradient occurs between black-fruited species (of both developmental patterns) and blue-fruited species, with the most extreme blue-fruited species being *V. davidii*. Short-wave cone stimulation (in the blue-region of the visible spectrum) provides the strongest differentiation between fruit color categories: red-fruited species have the lowest short-wave stimulation, followed by the black-fruited species of both developmental patterns, and finally blue-fruited species have the highest stimulation (Fig. 2). These color categories largely do not overlap in tetrahedral visual space: the only color categories with overlap > 0 are the two black-fruited color categories (Additional file: Figure S2). As a consequence, although there is a gradient between black and blue fruit colors, these categories are distinct when modeled in bird visual space.

Nutritionally, *Viburnum* fruits varied dramatically along an axis from high moisture and carbohydrates to high lipids and low moisture and carbohydrates. Moisture and carbohydrates ranged from 48 to 90% and 8–36% (34–93% dry mass), respectively (Fig. 2). Lipids constitute 0.2–22.8% (1.6–58% dry mass) of fruit pulp across species (Fig. 2). Protein content was low across all *Viburnum* species measured, ranging from 0.5–3.5% (1–9% dry mass); ash was similarly low, 0.3–4.4% (2–12% dry mass). Fruit length varied by a factor of > 3 across species, from a minimum of 3.5 mm (*V. propinquum*) to a maximum of 11 mm in length (*V. prunifolium*; Fig. 2). Pulp volume of the fruit varied from less than half (46%) to nearly all (92%) of the fruit (Fig. 2). Endocarp shape also varied enormously, from species that were extremely flat with an endocarp three times wider than thick (e.g., *V. opulus*) to species that were essentially round in cross-section (e.g., *V. tinus*).

### Fruit syndromes

**Fig. 3:**
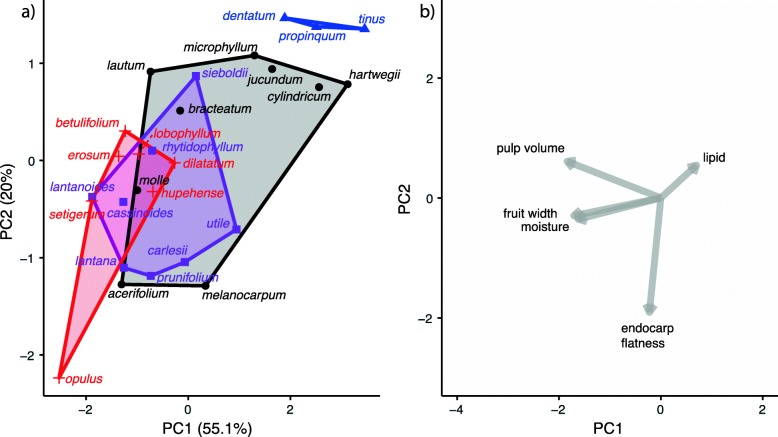
Phylogenetic PCAs of five fruit traits: lipid content, moisture content, pulp volume, fruit width, and endocarp flatness. The four fruit color categories identified here are plotted according to their colors: blue (triangles), red (+ marks), black-sequential (purple color, squares), and black-synchronous (black color, circles). **a** Convex hulls demonstrate that the fruit color categories differentiate in PCA space when accounting for phylogeny, even when color is not included as an input variable. **b** Variable loadings on the first two PC axes

Figure 3 shows a phylogenetic PCA based on five fruit traits. The first two PC axes explain 55.1% and 20.1% of the variance, respectively (Fig. 3). PC1 is positively correlated with having higher lipid content, a low pulp volume (i.e., endocarp filling much of the fruit), small fruit size, and low moisture content. PC2 is positively correlated with high lipid content, and negatively correlated with having a flattened endocarp. Fruits occur along an axis of watery fruits with low lipid content that tend to be red or black-sequential in color, to blue or black-synchronous fruits that are low in water, high in lipids, and have endocarps that occupy more of the volume for the fruit.

### Phylogenetic independent contrasts (PICs)

**Fig. 4:**
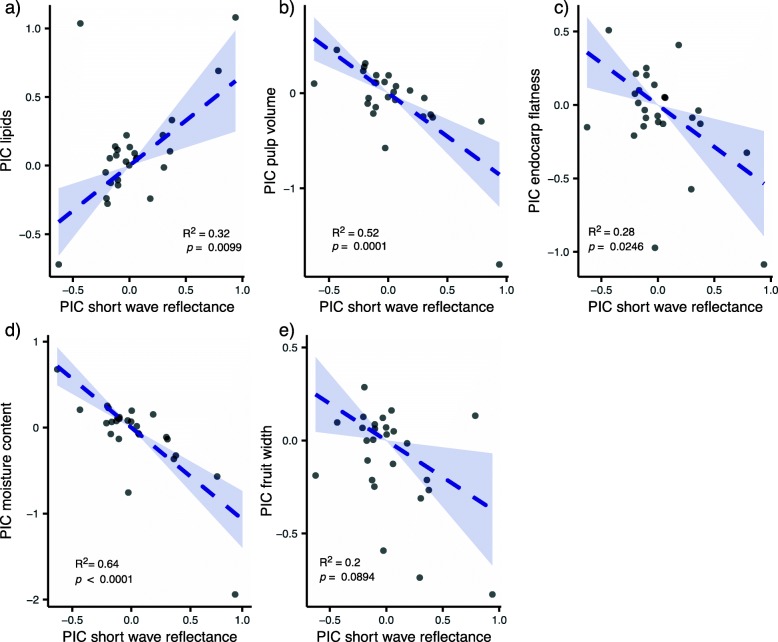
Phylogenetic independent contrasts (PICs) show that short-wave reflectance (blue-ish color) has evolved in correlation with lipid content, but not with other fruit traits. We illustrate here linear regressions with Bonferroni-adjusted *p*-values for all correlations between color (short wave reflectance) and the five fruit traits used in the phylogenetic PCA: **a** lipid content, **b** pulp volume, **c** endocarp flatness, **d** moisture content, and **e** fruit width. Blue ribbons indicate 95% confidence intervals

Pairwise regressions between PIC values of short-wave reflectance and the five fruit trait variables (lipid content, moisture content, pulp volume, endocarp flatness, and fruit width) indicate that color, especially a blue color, is correlated with higher lipid content (Fig. 4).

### Endocarp shape and fruit color correlations across Viburnum

**Fig. 5:**
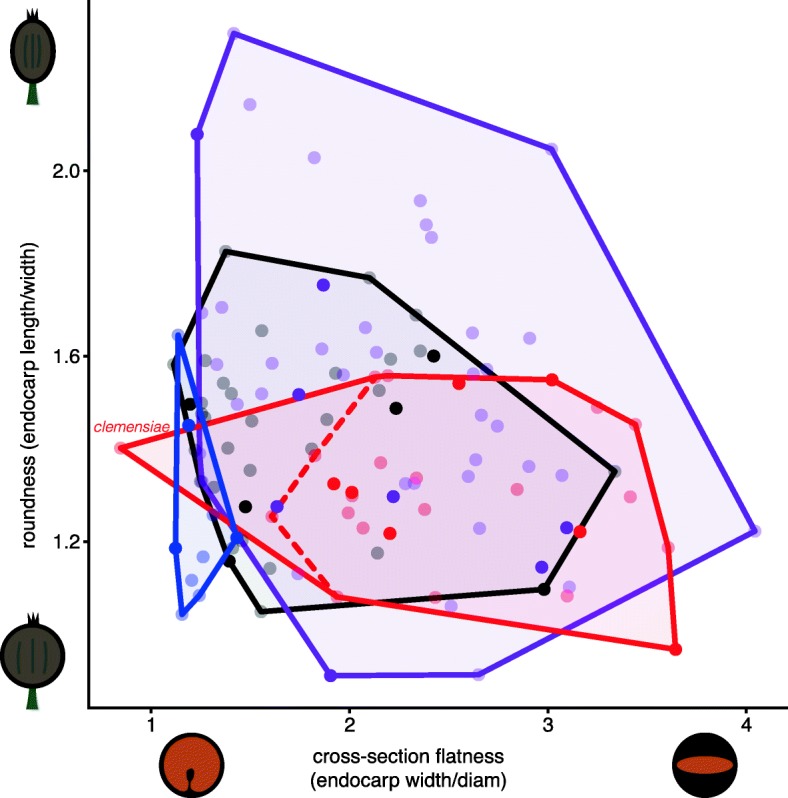
Blue and red fruits display different endocarp shapes across a broad range of *Viburnum* species (115 species). On the x-axis, we plotted endocarp shape in cross-section, where species with small values have rounder cross-sections while species with high values have flatter cross-sections. On the y-axis, we plotted endocarp shape in profile, where low values indicate round endocarps and high values indicate elliptical endocarps. Red-fruited species have flat endocarps, while blue-fruited species have rounder endocarps. The one exception is *V. clemensiae*, which is red but has a round endocarp (labeled to illustrate its position in endocarp shape space). The dashed red line shows the shape space for all red-fruited species excluding *V. clemensiae*. Opaque dots indicate species for which we generated new nutritional data in this paper

Across 115 species of *Viburnum*, there was not a statistically significant difference in the log of endocarp shape across fruit color categories (F = 1.66, *p* = 0.18; Fig. 5), even when we excluded the outlier *V. clemensiae* (F = 1.74, *p* = 0.16). However, our fruit color categories have non-equal variance (the blue-fruited color category has lower variance than any of the others; Brown-Forsythe test, F = 3.0, *p* = 0.03) as well as unequal sample sizes. As a consequence, we additionally used a non-parametric test, the Kruskal-Wallis test, which was significant (chi-squared = 37.0, *p* < 0.001), although this test does not incorporate phylogenetic relatedness. Overall, we find mixed evidence of a difference in endocarp shape across fruit color categories. Both black fruit types (black-sequential and black-synchronous) have variable endocarp cross-sections from flat to relatively round; black-sequential fruits, in particular, have explored nearly the entire endocarp shape space. We found little evidence of significant differences in endocarp shape viewed in the longitudinal (length) dimension across fruit color categories, with the exception that black-sequential fruits include a few species with more elliptical endocarps than in other color categories.

### Ancestral state reconstructions

**Fig. 6:**
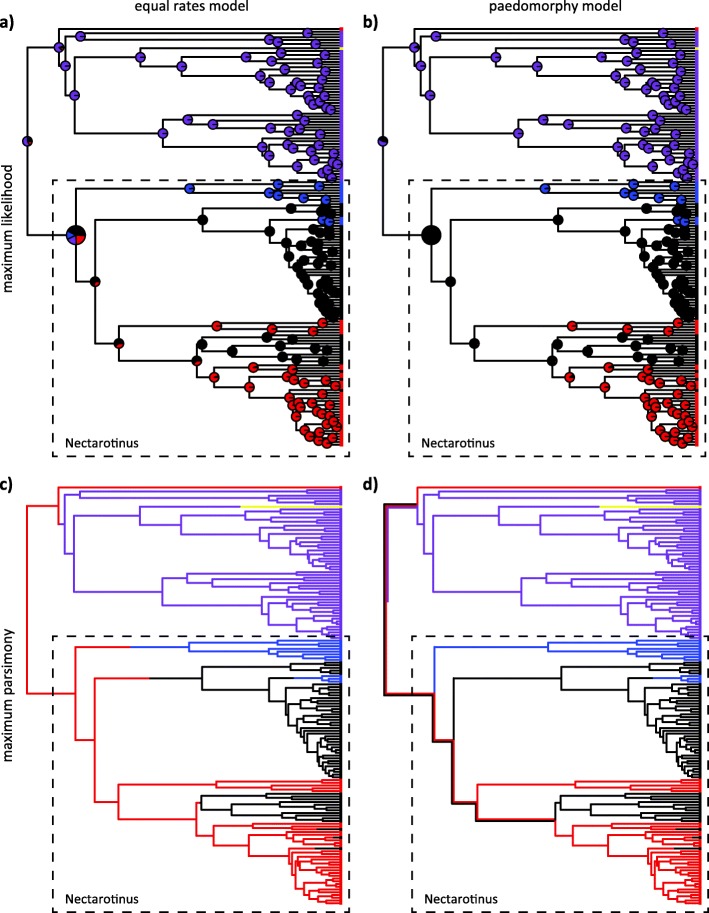
Ancestral state reconstructions of *Viburnum* fruit color, using maximum likelihood and parsimony and two different models of character evolution (equal rates/costs and paedomorphy). **a** Maximum likelihood reconstructs the ancestral state of Nectarotinus as most likely black-synchronous or red under the equal rates model, and as black-synchronous under the paedomorphy model. **b** Parsimony reconstructs the ancestor of Nectarotinus as red according to the equal rates model, but as black-synchronous or red according to the paedomorphy model. None of these analyses strongly support a paedomorphy hypothesis in which the ancestor of Nectarotinus was black-sequential. The Nectarotinus clade is indicated by the dashed line, and the colors of branches, tip labels, and node reconstructions indicate the fruit color category of the species (purple = black-sequential, black = black-synchronous, red = red-synchronous, blue = blue-synchronous)

**Fig. 7:**
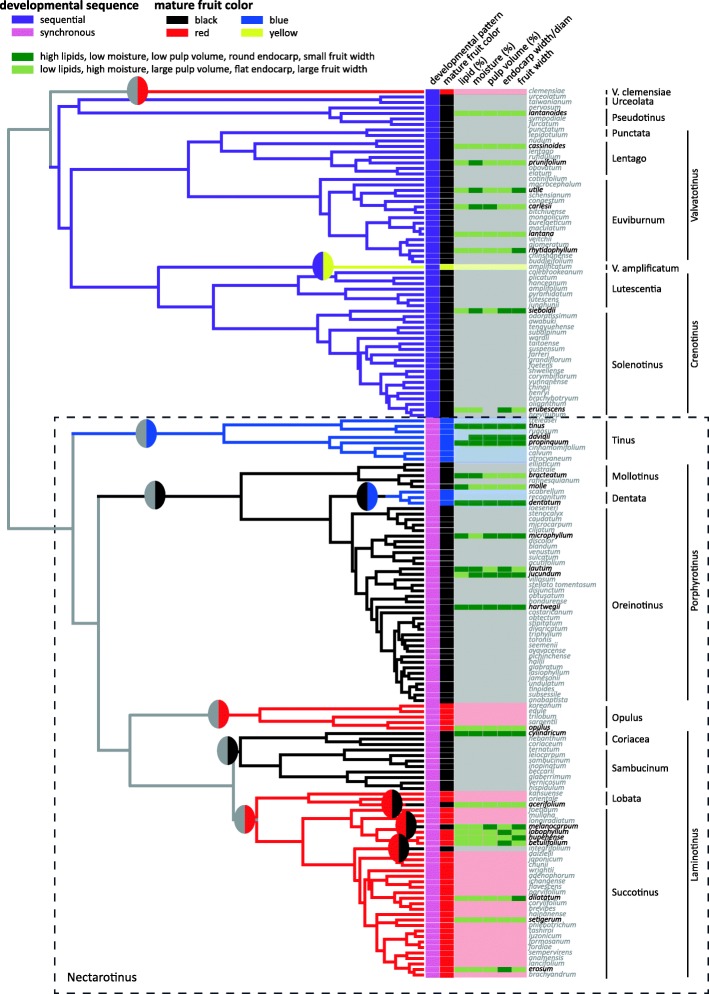
*Viburnum* fruits have evolved syndromes of traits, multiple times. Blue-fruited species have high lipid content, low moisture, low pulp content, and a rounder endocarp. Red-fruited species have extremely high moisture content and pulp content, low lipids, and a flatter endocarp. Illustrated here are the main traits we examined in this analysis, classified as to whether they resemble the blue-fruited syndrome or the red-fruited syndrome. We used the mean value of each trait as the threshold, and determined whether blue-fruited species had “high” or “low” values for that trait. Black-fruited species (both synchronously and sequentially developing) exhibit a range of traits, including some species with intermediate moisture levels that differ from those found in red-fruited or blue-fruited species. Gray branches indicate regions of the phylogeny where the ancestral state is especially equivocal

Stochastic character mapping estimated an average of ten transitions in fruit color across the *Viburnum* tree (Fig. 6; Fig. 7). One transition from black-sequential to red was inferred along the branch leading to *V. clemensiae*. Two transitions from black-synchronous to red were also identified, in 1) the Opulus clade, and 2) the Succotinus+Lobata clade. Three transitions from red to black-synchronous were also inferred, along the branches leading to 1) *V. melanocarpum*, 2) *V. integrifolium*, and 3) *V. acerifolium.* Two transitions from black-synchronous to blue were inferred. One of these occurred along the branch leading to the Tinus clade. The second occurred along the branch leading to the *V. dentatum* complex. A single shift from black-sequential to yellow was inferred along the branch leading to *V. amplificatum*. Finally, a shift from black-sequential to black-synchronous occurred early in Nectarotinus evolution, but the branch on which this transition occurred is uncertain. Due to the uncertain location of this transition, in some reconstructions multiple transitions from black-sequential to black-synchronous are inferred in order to explain the present-day distribution of traits. Seven of the nine transitions with relatively certain locations occurred in the large Nectarotinus clade; the only exceptions are the transitions to red in *V. clemensiae* and from black-sequential to yellow in *V. amplificatum*.

Our maximum likelihood models favored the ancestor of *Viburnum* as most likely exhibiting a black-sequential (indicated in purple in Fig. 6) fruit color, but we note that this is equivocal. In contrast, our parsimony models favored a red-, black-synchronous, or black-sequential ancestor. This root state is thus ambiguous. As discussed further below, the condition in the ancestor of the Nectarotinus clade (Fig. 7) is critical in assessing the role of heterochrony in *Viburnum* fruit evolution, as most of the fruit color diversity (and hence most of the potential for paedomorphic evolution) occurs in this clade. We find that the ancestral state of Nectarotinus depends strongly on the model of character evolution. The best model of evolution under maximum likelihood was the equal rates model (where all transitions are allowed), and reconstructs Nectarotinus as most likely black-synchronous, with red-synchronous as the second most likely state (black-sequential also has moderate support) (Fig. 6a). Under parsimony, the backbone of Nectarotinus is most likely red (when transitions are unconstrained; Fig. 6c). Under our paedomorphy model, maximum likelihood infers that the ancestor of Nectarotinus was almost certainly black-synchronous, while parsimony reconstructs that this ancestor was either black-synchronous or red (Fig. 6b, d).

## Discussion

We find strong support for the existence of fruit syndromes in *Viburnum*, with significant correlations between fruit color, morphology, and nutritional content (summarized in Fig. 7). We identify two strong syndromes (in blue- and red-fruited species) as well as a weaker syndrome (black-sequential) and one fruit color category with high variability (black-synchronous). Blue fruits are high in lipids, with low moisture content and relatively small, round endocarps. Red fruits exhibit traits on the opposite end of the spectrum: they tend to have low lipid content but very high moisture, and are larger in size with a flatter endocarp. These two syndromes likely both evolved from black-synchronous ancestors (although black-sequential is also a possibility; see below). Species with black-synchronous fruits do not form a single clade and, not surprisingly, exhibit the highest variability. Some resemble red-fruited species, including *V. acerifolium* and *V. melanocarpum*. This makes sense as these species appear to have evolved quite recently from red-fruited ancestors. Other black-synchronous species resemble their blue-fruited relatives in having high lipid content and large, round endocarps. Black-sequential fruits most resemble red fruits in having a relatively high moisture content and low lipid content. However, unlike red fruits, black-sequential fruits vary enormously in the shapes of their endocarps, from very flat (e.g., *V. lentago*) to round (e.g., *V. sieboldii*).

The differences between red and blue fruits are particularly striking. Blue fruits are lipid-rich with a low moisture content and have a large, round endocarp that takes up much of the fruit’s volume, leaving only a small quantity of nutritious pulp. This syndrome evolved twice, once in the Tinus clade and once in Dentata. Relatives of *V. dentatum* in the Porphyrotinus clade (e.g., *V. molle, V. bracteatum,* and *V. lautum*) have high lipid content (7.3–12.5% [22–35% dry mass] with the exception of *V. jucundum* with only 3.7% [9% dry mass]), which suggests that high lipid content evolved prior to the origination of blue fruit color in *V. dentatum*. Because blue fruits in *Viburnum* embed lipids in their cell walls to produce a structural color [58, 59], the evolution of lipid-rich pulp prior to the evolution of a blue fruit color suggests that the up-regulation of lipid synthesis may have set the stage for the subsequent use of lipid droplets in the production of structural color. All species in the Tinus clade have blue fruits and likely have high lipid content, so in this case the order in which these traits evolved is unclear.

Across 115 species of *Viburnum*, mature fruit colors occupy distinct regions of endocarp shape space that correspond with the syndromes described here: blue fruits have rounder endocarps, red fruits have flattened endocarps, and both black fruit types (sequential and synchronous) span nearly the whole range of endocarp shapes. The only exception to this is the red-fruited *V. clemensiae*, which has a round endocarp more similar to blue-fruited species. The fruits of *V. clemensiae* are poorly known and only a handful have ever been collected, but analyzing their nutritional content would provide further insight into the evolution of fruit syndromes in *Viburnum.* Aside from *V. clemensiae*, red fruits are juicy, with high moisture content, low lipid content (and corresponding high carbohydrate content), and a flattened endocarp that takes up a small portion of the total volume of the fruit. This syndrome evolved twice, once in the Opulus clade and once in the Succotinus+Lobata clade. It is especially well developed in the Opulus clade, where the fruits have exceptionally flattened (nearly ungrooved) endocarps [32].

The selective pressures driving *Viburnum* fruit evolution are unknown, but may relate to disperser characteristics. For instance, migratory birds may consume lipid-rich fruits in order to meet their daily energy requirements, as lipid content is positively correlated with a fruit’s energy density [3, 33, 60, 61]. This energy-dense fruit comes with a high cost, however: the bird must carry the weight of the endocarp in its gut until defecation. In the Mediterranean, preferential consumption of lipid-rich fruits during winter by European robins (*Erithacus rubecula*) has been noted [62]. Similar patterns have been reported in eastern North America, where lipid-rich fruits (including *V. dentatum*) are consumed by migratory birds more frequently than are less lipid-rich species [33, 34, 63]. However, birds are rarely observed consuming *only* lipid-rich fruits, and instead they switch between complementary food sources where some food items play a major role in their diet and others a minor role [21, 64]. Red, juicy fruits appear to target a different feeding strategy and offer a low-quality reward (mostly water, a little sugar) at a low cost (a small endocarp). These two syndromes thus represent two distinct strategies: high value, high cost, combined with a highly distinctive color in the case of blue fruits, and low value, low cost, and a common color in the case of red fruits.

Dispersers do appear to choose fruits based on the relative ratio of reward and cost [65]. A large, round endocarp — a higher cost to a bird — may have been enabled in blue-fruited lineages in part because of the high value of the lipids, which are energy dense and relatively rare in fruits [66]. Because blue-fruited species offer a high-quality reward, they are able to produce a higher cost and still be dispersed. Black-fruited species reflect a similar, though weaker, pattern. Species with larger, rounder endocarps — e.g., black-synchronous species such as *V. hartwegii, V. jucundum,* and *V. lautum,* as well as black-sequential species such as *V. sieboldii* — also tend to have elevated lipid levels. Black-sequential species with flatter endocarps (e.g., *V. prunifolium, V. lantana*) have very low lipid content, similar to lower quality red-fruits, which would likely be consumed only rarely if they did not offer a larger quantity of reward.

Broadly speaking, fruit colors in *Viburnum* — especially blue and red — appear to act as honest signals: blue color always occurs with higher-value, lipid-rich pulp, while red color is associated with larger quantities of water- and sugar-rich pulp. In addition to inferring something about the nutritional content of a *Viburnum* fruit based on its color, dispersers can also infer that a blue fruit will have a higher cost than a red fruit in terms of endocarp size relative to the quantity of pulp. The overall value of consuming any individual fruit depends on the quantity and value of the reward as well as on the size of the endocarp.

### Evolutionary origins of fruit color diversity

To better understand the evolutionary origins of fruit color diversity in *Viburnum*, we conducted a series of ancestral state reconstructions meant to assess whether paedomorphic evolution can explain *Viburnum* fruit color diversity. The root ancestral state of *Viburnum* is equivocal, and was likely either black-sequential (as suggested by maximum likelihood) or red (as suggested by parsimony), although black-synchronous is also possible. Most of our models prefer a black-fruited ancestor, either sequentially or synchronously developing. Paedomorphic evolution, if true, would suggest that the ancestor to *Viburnum* likely experienced a series of color stages during development that were subsequently modified. However, the topology of the *Viburnum* tree makes it difficult to confidently infer the ancestral state, and without alternative lines of evidence we cannot say with any degree of certainty what fruit color category and developmental pattern this ancestor displayed. The sister group to *Viburnum* includes *Sambucus* (elderberries), which also produces fleshy fruits. Phylogenetic analyses suggest that black fruits are ancestral within *Sambucus*, and red fruits are derived [67]. This lends some support to the view that mature black fruits are ancestral in *Viburnum*, but it does not help with the developmental pattern, as these have not been well documented in *Sambucus*.

The equivocal nature of our reconstructions is unsurprising, because *Viburnum* undergoes a bifurcation early in its evolution. One major branch exhibits almost entirely black-sequential fruit development, while the other exhibits a wide range of fruit colors. In one of these clades (including *V. clemensiae*, Valvatotinus, Crenotinus, Pseudotinus, and Urceolata) the fruits develop sequentially. Our reconstructions favor black-sequential as the ancestor of this clade, though red is also a possibility according to parsimony. As far as is known, all species except two retain this presumed ancestral condition. We note, however, that in spite of decades of research on *Viburnum* there are species whose fruit development is still poorly known (e.g., the two *Punctata* species). There is also considerable variation in the realization of this fruit color category. In some species the red phase is very short and black fruits persist for many months (e.g., *V. lentago*), while in others the red phase is prolonged and the fruits turn black (and juicy) only shortly before dispersal (e.g., *V. erubescens*). The two species within this clade known to have substantially modified this developmental program are *V. clemensiae* and *V. amplificatum*. In both species, paedomorphic evolution is possible although would need to be further studied to be confirmed. *V. clemensiae*, to the best of our knowledge, has red fruits at maturity, which could have evolved from a loss of the final, mature black stage. *V. amplificatum* matures exceptionally large yellow fruits that could potentially be mammal-dispersed (perhaps by orangutans, which occupy the same forests). As with *V. clemensiae*, this condition could possibly have evolved by the loss of the final red and black stages.

In the other major clade (Nectarotinus), our models disagree as to the ancestral state. This is the clade within which fruit color diversification seems to have occurred at a higher rate, driven possibly by paedomorphic evolution or by some other process. Seven of the nine confidently inferred fruit color transitions occurred in Nectarotinus; only two transitions occurred in the other major clade. According to our models, the ancestor to Nectarotinus was most likely black-synchronous, but could have been red, black-sequential, or, less likely, even blue. The difficulty of confidently reconstructing ancestral states reflects the early bifurcation of the *Viburnum* phylogeny, one with nearly all the same fruit color category (black-sequential) and one (Nectarotinus) with a wide variety of fruit color categories. This topology is not amenable to confident ancestral state reconstructions, and alternative lines of evidence would be needed to bear more strongly on this question. There is an alternative topology for the *Viburnum* phylogeny than the one we have used here; most phylogenetic analyses have placed *V. clemensiae* as sister to all remaining *Viburnum,* rather than as sister to one of the two major clades [22, 24]. However, this alternative topology does not alter the ancestral state reconstructions significantly. With *V. clemensiae* placed sister to the rest of *Viburnum,* we would have three lineages, each with a different fruit color category: one red-fruited, one black-sequential, and one possibly black-synchronous although equivocally so. Overall, we do not find strong support for the paedomorphy hypothesis, which would only be favored if this ancestor were confidently reconstructed as black-sequential.

The equivocal nature of these reconstructions suggests that perhaps the ancestor to Nectarotinus did not fall neatly into either of the two categories that we have used to describe extant black fruits, black-sequential and black-synchronous, and instead was a non-analog fruit type that exhibited both synchronous development and the immature color phases. Synchronous fruit development clearly evolved along the branch subtending Nectarotinus. However, immature color phases may not have been lost along the same branch, and evidence from extant species supports this possibility. We have broadly characterized synchronously fruiting species as lacking immature color phases, but this is not strictly correct. Some extant species in Nectarotinus do exhibit immature red colors. For instance, *V. ellipticum* has a clear red phase but the fruits mature relatively synchronously [56]. *Viburnum tinus* and *V. cylindricum* sometimes exhibit red phases, though to a much lesser extent and also with considerable variation among individuals. If the ancestor to Nectarotinus had a more pronounced red phase than we find in synchronous species today, the immature red phase in this non-analog ancestral fruit type would have been lost or greatly reduced multiple times (in Tinus, Porphyrotinus, and Coriacea + Sambucina). And, if enough of a red phase persisted in the ancestor of Nectarotinus, this could have provided the basis for paedomorphic evolution of red fruits in the Opulus and Succotinus+Lobata clades. The Opulus and Succotinus clades display additional attributes that are consistent with their fruits having been juvenilized. For instance, they tend to hang on the plant for long periods of time after maturing [68], and are relatively unpalatable [69, 70] — both traits that are characteristic of a juvenile fruit that needs to remain attached and uneaten.

If the ancestor to Nectarotinus likely did not have a fully sequentially developing fruit, can paedomorphic evolution nonetheless explain the variety of fruit syndromes within Nectarotinus? One possibility is that the shift to synchronicity along the Nectarotinus branch enabled the subsequent diversification of *Viburnum* fruit colors via paedomorphic evolution. For a sequentially developing fruit to undergo paedomorphy, selection for fruit colors would have to be strong and consistent over much of the maturation period, which often lasts for several months in *Viburnum.* Synchronously developing *Viburnum* fruits, on the other hand, tend to fruit for shorter periods of time during the late summer and fall. For these fruits to evolve paedomorphically, migratory birds would need to arrive only a short period of time before fruits were fully mature. At that point, a mismatch in the timing of maturation and bird migration of a few days or weeks could drive birds to consume immature fruits and consequently to select for fruit maturation at an earlier color stage. If the ancestor to Nectarotinus were a non-analog fruit type that developed synchronously and over a short period of time, but retained an immature red phase, even small mismatches in timing could have promoted paedomorphic evolution.

Regardless of whether such paedomorphic evolution drove fruit color diversification, synchronicity may represent a fundamental shift in strategy away from targeting residential birds and towards targeting migratory and/or flocking birds. For the most part, sequentially developing species fruit over weeks or even months (cf. the temporally bicolored fruits described by [71–73]) and potentially target summer resident birds as their primary dispersers. Birds are able to select mature fruits out of mixed displays, and may use information about the relative ratio of immature to mature fruits to choose which fruits to consume [74]. Sequentially developing species advertise their future ripe fruit, in addition to clearly identifying currently ripe fruit, through the temporal contrast between immature fruit colors and mature fruit colors.

In contrast to sequentially developing species, synchronously developing species may target flocking and/or migratory birds. In the synchronously fruiting *V. dentatum,* larger crop sizes are associated with higher rates of removal by flocking bird species [75], although fruits are removed more slowly when individuals occur in large clumps [76]. These different strategies — should they hold up to future research — may also explain the great diversity of fruit colors in the Nectarotinus clade. The need to maintain multiple color stages may limit the rate of evolution in sequentially developing species, and this may extend beyond just the mature color of the fruit to nutritional traits. Sequentially developing species retain similar nutritional content (relatively low lipids, high carbohydrates, and intermediate moisture), though they do vary in their endocarp shapes. As a consequence, sequentially developing species, by and large, have not deviated significantly from their presumed ancestral fruit. At this point it is impossible to say whether it is purely a result of phylogenetic accident that this large clade does not appear to have explored trait space very much, or whether there are constraints that make deviation from this fruit unsuccessful. But we find it intriguing that sequentially developing fruits exhibit relatively little variance in their traits, while synchronously developing fruits exhibit a much greater diversity in fruit color, nutrition, and morphology.

Synchronously developing species are not constrained by the need to display immature and mature colors simultaneously and thus are free to evolve in response to other selective pressures. For instance, they may evolve distinctive colors in order to more effectively compete for dispersers, or mimic higher quality fruits [20, 77]. We see this possibility in the blue-fruited syndrome. Sequentially developing fruits may reduce competition for dispersers by spreading their fruiting out over a longer period of time [78], while mast fruiting in synchronously developing fruits may be advantageous in relation to attracting migratory and/or flocking birds that consume large quantities of fruit at once [33, 34, 63]. At the same time, synchronicity might also promote the evolution of specialized fruit colors and nutritional content.

Our ancestral state reconstructions and transition rate analyses support the notion that rates of trait evolution have been higher in Nectarotinus. When we estimate transition rates between each fruit color, we find that the highest transition rates are between red and black-synchronous (Additional file 1: Table S4). The high rate of red to black-synchronous transitions is driven by the three independent origins of black-synchronous fruits from red-fruited ancestors, all of which happened relatively recently (*V. acerifolium* in the Lobata clade, and *V. melanocarpum* and *V. integrifolium* in Succotinus). Black-sequential fruits have non-zero transition probabilities to all color categories except blue, although these rates are very low. Black-sequential fruits are also the only color category with non-zero transition probability to yellow, which is unsurprising as there is only one modern species with yellow fruits at maturity (*V. amplificatum*) and it appears to have evolved from a black-sequential ancestor. We note, however, that yellow-fruited forms of red-fruited *Viburnum* species are quite common (e.g., *V. opulus* f *xanthocarpa* in the Opulus clade; *V. dilatatum* var. *xanthocarpum* and *V. phlebotricum* f. *xanthocarpum* in Succotinus; see [31]). Because seven out of the nine identified fruit color transitions occur within Nectarotinus and this ancestor is frequently reconstructed as black-synchronous, black-synchronous shows moderate transition rates to both blue and red fruit colors. These results further emphasize the difference between black-sequential fruits, which exhibit very low rates of trait evolution, and the faster rates of evolution that occur within synchronously developing fruits.

### Biogeography and dispersal

The biogeography of fruit colors relates not only to their phylogenetic history, but also to fruit traits that may facilitate dispersal. It is noteworthy that *Viburnum* fruits within particular biogeographic regions tend to complement one another. For example, in eastern North America, native *Viburnum* species include both the lipid-rich blue-fruited *V. dentatum*, the carbohydrate-rich black-sequential *V. lantanoides*, the black-synchronous *V. acerifolium,* and the red-fruited *V. trilobum*. Europe’s native *Viburnum* flora includes a lipid-rich blue-fruited species (*V. tinus*), a black-sequential species (*V. lantana*), and two red-fruited species (the widespread *V. opulus,* as well as *V. orientale* in the Caucasus mountains of Georgia). Europe is missing only the black-synchronous fruit syndrome. Asia is the center of both species and phylogenetic diversity in *Viburnum,* and each of the fruit syndromes is also represented in that region. Thus, across most of the biogeographic range of *Viburnum,* all or nearly all of the syndromes are present and often represented by multiple species.

However, two regions have unusual *Viburnum* color communities. In the mountains of the Neotropics, there are only two fruit color categories, black-synchronous fruits with rounded endocarps in ~ 36 species of the Oreinotinus clade and a single black-sequential species, *V. elatum*, in Mexico. This unusual *Viburnum* color community simply reflects the fact that only the Oreinotinus clade successfully radiated into the cloud forests of the Neotropics [24, 26]. The second region with a somewhat unusual *Viburnum* color community is tropical Southeast Asia, which lacks a significant presence of red-fruited species. *Viburnum clemensiae* inhabits tropical forests in northern Borneo, and only the widespread *V. luzonicum* of the red-synchronous Succotinus clade extends south into the mountains of the Philippines.

These fruit color communities in *Viburnum* have been assembled largely through the movement of clades around the Northern Hemisphere rather than through the evolution fruit colors in situ*.* For example, there appear to have been six movements from the Old World into North America [22]. In four of these cases, plants arrived with fruit syndromes that had evolved earlier in Asia. Members of the Lentago clade and *V. lantanoides* (Pseudotinus) retained black-sequential fruits, while *V. edule* and *V. trilobum* (two separate entries into North America within the Opulus clade) retained their ancestral red fruits. It is unclear whether the large Porphyrotinus clade entered the New World with black-synchronous fruits or whether they evolved this fruit type upon arrival. The most interesting case in North America is *V. acerifolium,* which is descended from red-fruited ancestors in the Old World (its closest relatives are *V. orientale* in the Caucusus mountains and *V. kansuense* in China). It may have evolved a black fruit color in the Old World and then become extinct there after moving to North America, but more parsimoniously it evolved black-synchronous fruits after entering North America (possibly mimicking the black fruits of Porphyrotinus species, which were already present at that time in North America). Multiple cases of dispersal to a new region followed by fruit color evolution have been documented in other angiosperms; e.g., from red to black in Gaultherieae [79] and from black to red in *Empetrum* [80]. Our analyses show that this has been rare in *Viburnum*.

Another case of convergence may relate, in quite a different way, to geography. So far as we know, no member of the Eurasian Tinus clade (with blue lipid-rich fruits) ever entered the New World, leaving room for the independent evolution of this syndrome in that region. This niche may then have been filled by *V. dentatum*, which evolved blue color in a clade that already had evolved lipid-rich fruits. Both the *V. tinus* species complex in Europe (which includes a species endemic to the Canary Islands, *V. rugosum*, and another to the Azores, *V. treleasii*), and the *V. dentatum* complex in Eastern North America, have exceptionally broad geographic ranges and large populations sizes. In both cases, it is plausible that blue lipid-rich fruits, broad geographic ranges, and large population sizes relate to dispersal by migratory bird species [33, 34, 62, 76].

Recent work on *Gaultherieae* [79] also addressed fruit color evolution, focusing on links between biogeography and fruit color. Their findings suggest that different fruit colors may have different propensities for dispersal, specifically that red fruits are more likely to disperse long distances and then evolve new fruit colors in situ*.* Unlike in *Gaultherieae*, we find only a single case of dispersal followed by in situ fruit color evolution, that of *V. acerifolium* (see above). However, as in *Gaultherieae,* it is possible that red-fruited lineages are more likely to disperse long distances than would be expected by chance. Of roughly twelve inter-continental dispersals in *Viburnum* [22], six of these occurred in red-fruited lineages while only a quarter of *Viburnum* species exhibit red fruits. Although the pattern is not as strong in *Viburnum* as it is in *Gaultherieae,* it does support the notion that red fruits may undergo long distance dispersal more frequently than other fruit colors. If this is true, it may help to explain other broad spatial patterns, such as the prevalence of red fruits at high latitudes in areas that have been recently recolonized following Pleistocene glaciations [81].

## Conclusions

Careful studies of the factors that underlie fruit trait diversification in particular lineages will help to advance our understanding of fruit evolution more broadly. Here, we have focused on correlated evolution between color, nutritional content, and morphology — the entire “package” that dispersers interact with. The syndromes we identify, as well as the potential significance of developmental trajectory (i.e., sequential vs. synchronous development), highlight that there are many under-explored aspects of fleshy fruit diversity. We suspect that syndromes of fruit traits, independent of the major dispersers (birds and mammals), will be found in other fleshy-fruited lineages. *Viburnum* fruits, with the exception of the unusual blue structural color in two lineages, are hardly unique: their nutritional contents, colors, sizes, and developmental patterns fall well within the range observed on a global scale [66]. Yet we have little reason to expect that the syndromes we have discovered in *Viburnum* will be identical in other lineages. If these syndromes were universal, all red fruits would have the same set of traits as red *Viburnum* fruits, which is clearly not the case. As we noted at the outset, correlations between color, nutritional content, and morphology differ across communities/regions [13, 18, 82]. Clearly, the scale at which syndromes are assessed is crucial: individual clades may exhibit strongly marked syndromes of fruits traits (as we have documented in *Viburnum*), but if these syndromes differ from one clade to another such patterns may be obscured at the community level.

The ecological and evolutionary consequences of the shift between sequential and synchronous fruit development discussed here is speculative, and it would be valuable to test this hypothesis in the many other temperate shrubs and trees that exhibit similar patterns of fruit color variability (e.g., *Rubus, Ribes, Vaccinium, Cornus*, etc.). Our observations in *Viburnum* suggest that 1) sequentially developing species might tend to be dispersed by resident birds while synchronously fruiting species might tend to be dispersed by flocking and/or migratory birds; 2) that synchronously fruiting species mature for short periods of time during the peak migration of birds through that region while sequentially developing species mature over longer periods of time; and 3) that synchronously developing clades exhibit faster rates of trait evolution than sequentially developing clades. These patterns may hold in other lineages and sequential versus synchronous fruit development may be a key factor underlying fruit trait diversification on a global scale.

## Supplementary information

**Additional file 1: Table S1.** Nutritional, morphological and (categorical) color data for *Viburnum* species, as well as accession numbers and sample sizes for each measurement. **Table S2.** Step cost matrix implemented in Mesquite to represent the paedomorphy model. **Table S3.** Transition rate matrices used in the ancestral state reconstruction by maximum likelihood. **Table S4.** Transition rates between each fruit color state as estimated by make.simmap in *phytools* using a symmetrical model of evolution.. **Figure. S1.** Species’ colors plotted in tetrahedral color space according to a visual model of a UV-sensitive bird. **Figure S2**. Volume overlap between each fruit color category in tetrahedral color space.

## Data Availability

All data generated in this study are made publicly available at Data Dryad: 10.5061/dryad.h44j0zpft.

## Abbreviations

AICc: Akaike information criterion (corrected)
ANOVA: Analysis of variance
AOAC: Association of Official Analytical Chemists
PCA: Principal components analysis
PICs: Phylogenetic independent contrasts
RAD-seq: Restriction-site associated DNA sequencing
