# Supplementary material for: Fruit syndromes in Viburnum: correlated evolution of color, nutritional content, and morphology in bird-dispersed fleshy fruits
Source: BMC Evol Biol. 2020 Jan 13;20:7. doi: 10.1186/s12862-019-1546-5 (PMC6956505; doi:10.1186/s12862-019-1546-5)

ADDITIONAL FILE

**Table S1.** Nutritional, morphological and (categorical) color data for *Viburnum* species, as well as accession numbers and sample sizes for each measurement. For lipids, protein, ash, and carbohydrates, we show the percent fresh mass with the percent dry mass in parentheses, followed by the number of replicates per measurement. Pulp volume was calculated based on the ratio of endocarp volume to fruit volume. For plants accessioned at botanical gardens and arboreta, we included the accession number for the living plant. For street plants not grown in botanical gardens, we included the specimen number for the voucher deposited in at the herbarium of the Yale Peabody Museum. Codes are as follows: AA = Arnold Arboretum, CUBG = Cambridge University Botanical Garden, UCBG = University of California at Berkeley Botanical Garden, YU = Yale University Herbarium, VL = voucher lacking. All samples were derived from fresh fruit material from living plants, not from the herbarium specimens. In several species, insufficient fresh fruit material was available to quantify all nutritional information (since quantification involves destruction of fruit material). Due to missing protein data, we did not include this factor in our phylogenetic PCA. Fruit colors are categorized as black-synchronous, black-sequential, blue, or red.

| **Species** | **Herbarium/garden** | **Collector code & number** | **Fruit color category** | **Moisture (%)** | **Lipid (%)** | **Protein (%)** | **Ash (%)** | **Carb (%)** | **Pulp volume (%)** | **Endocarp width/diam** |
| --- | --- | --- | --- | --- | --- | --- | --- | --- | --- | --- |
| *V. acerifolium* | YU | MAS 506 | black (syn) | 86 (n=3) | 0.6 (4.2; n=3) | 0.5 (3.7; n=3) | 1.7 (11.7; n=3) | 11 (80) | 87 | 2.75 (n=20) |
| *V. betulifolium* | AA | 1964-80-A | red | 90 (n=3) | 0.6 (6.3; n=3) | 0.6 (6.1; n=2) | 0.4 (4.2; n=3) | 8 (83) | 87 | 1.71 (n=20) |
| *V. bracteatum* | AA | 1068-87-A | black (syn) | 64 (n=1) | 12.5 (35.2; n=1) | NA | 3.5 (9.8; n=1) | NA | 84 | 1.94 (n=7) |
| *V. carlesii* | YU | MAS 501 | black (seq) | 70 (n=3) | 0.5 (1.8; n=3) | 1.2 (4.0; n=3) | 0.8 (2.6; n=3) | 28 (92) | 76 | 2.5 (n=20) |
| *V. cassinoides* | AA | 593-2008-A | black (seq) | 82 (n=3) | 1.2 (6.5; n=3) | 0.7 (3.9; n=2) | 0.6 (3.3; n=3) | 15 (86) | 90 | 2.3 (n=20) |
| *V. cylindricum* | CUBG, RBG | 2004-958 (RBG); 19630030*A (CUBG) | black (syn) | 61 (n=2) | 22.8 (58.2; n=2) | 1.3 (3.3; n=2) | 1.6 (4.2; n=32) | 13 (34) | 65 | 1.9 (n=20) |
| *V. davidii* | VL | MSA, 11/1/18, Cambridge, UK, S.N. | blue | 53 (n=1) | NA | NA | 2.3 (4.8; n=1) | NA | 48 | 1.1 (n=7) |
| *V. dentatum* | AA | 5070-1*C | blue | 57 (n=4) | 17.1 (39.6; n=3) | 1.8 (4.1; n=3) | 4.4 (10.1; n=4) | 20 (46) | 66 | 1.23 (n=20) |
| *V. dilatatum* | AA | 1834-77-E | red | 88 (n=3) | 0.7 (5.9; n=3) | 0.6 (5.2; n=3) | 0.5 (3.8; n=3) | 11 (85) | 81 | 1.93 (n=20) |
| *V. erosum* | AA | 619-88-A | red | 90 (n=3) | 1 (10.1; n=3) | 0.5 (5.2; n=3) | 0.6 (5.7; n=3) | 8 (79) | 87 | 1.86 (n=20) |
| *V. erubescens* | AA | 798-65-A | black (seq) | 84 (n=2) | 2.6 (16.2; n=1) | 1.5 (9.3; n=2) | 1.0 (6.0; n=2) | 11 (69) | NA | 1.73 (n=20) |
| *V. hartwegii* | VL | MJD, 2/4/19, Chiapas, Mex., S.N. | black (syn) | 55 (n=2) | 9.4 (20.8; n=2) | 1.5 (3.4; n=2) | 1.5 (3.4; n=2) | 33 (72) | 50 | 1.24 (n=20) |
| *V. hupehense* | AA | 80-81-C | red | 90 (n=3) | 0.9 (9.0; n=3) | 0.6 (5.4; n=3) | 1.0 (9.8; n=3) | 8 (76) | 81 | 2.02 (n=20) |
| *V. jucundum* | VL | MJD, 2/4/19, Chiapas, Mex., S.N. | black (syn) | 56 (n=2) | 3.7 (8.3; n=2) | 1.8 (4.0; n=2) | 2.6 (5.9; n=2) | 36 (82) | 66 | 1.28 (n=20) |
| *V. lantana* | AA | 206-96-B | black (seq) | 81 (n=3) | 0.4 (2.3; n=3) | 1.6 (8.4; n=3) | 1.7 (8.9; n=3) | 15 (80) | 85 | 2.58 (n=13) |
| *V. lantanoides* | VL | MSA, 11/2/2019, Lee, MA, S.N. | black (seq) | 87 (n=1) | 0.2 (1.6; n=1) | NA | 1.5 (11.0; n=1) | NA | 88 | 2.05 (n=8) |
| *V. lautum* | UCBG | 76.2051 | black (syn) | 73 (n=3) | 8.3 (30.6; n=3) | 0.8 (2.9; n=2) | 2.3 (8.6; n=3) | 16 (58) | 84 | 1.48 (n=20) |
| *V. lobophyllum* | AA | 1875-80-A | red | 89 (n=2) | 0.3 (2.6; n=2) | 0.9 (7.7; n=2) | 1.1 (9.7; n=2) | 9 (80) | 83 | 1.78 (n=20) |
| *V. melanocarpum* | AA | 386-81-D | black (syn) | 85 (n=3) | 1.9 (12.9; n=3) | 0.7 (5; n=3) | 1.5 (10.2; n=3) | 11 (72) | 66 | 2.42 (n=20) |
| *V. microphyllum* | YU | MJD, 3/20/19, Oaxaca, Mex., S.N. | black (syn) | 76 (n=2) | 6 (25.1; n=2) | 2.2 (9.1; n=1) | 0.9 (3.7; n=2) | 15 (62) | 64 | 1.04 (n=10) |
| *V. molle* | AA | 167-2000-A | black (syn) | 77 (n=3) | 7.3 (32.5; n=3) | 1 (4.6; n=3) | 1.4 (6.1; n=3) | 13 (57) | 88 | 2.35 (n=20) |
| *V. propinquum* | RBG | 2007-1324 | blue | 58 (n=1) | 6.7 (16; n=1) | 3.5 (8.5; n=1) | 1.7 (4.0; n=1) | 30 (72) | 69 | 1.3 (n=20) |
| *V. prunifolium* | YU | MAS 502 | black (seq) | 65 (n=2) | 0.6 (1.7; n=2) | 0.8 (2.3; n=1) | 0.9 (2.6; n=2) | 33 (93) | 84 | 2.74 (n=16) |
| *V. rhytidophyllum* | AA | 1387-82-B | black (seq) | 81 (n=3) | 0.5 (2.8; n=3) | 0.8 (4.3; n=3) | 1.9 (10.2; n=3) | 16 (83) | 89 | 2.01 (n=20) |
| *V. setigerum* | AA | 305-2002-A | red | 89 (n=3) | 0.9 (8.2; n=3) | 0.5 (4.5; n=3) | 0.3 (3.1; n=3) | 9 (84) | 92 | 2.22 (n=20) |
| *V. sieboldii* | YU | MAS 505 | black (seq) | 74 (n=1) | 3.2 (12.2; n=1) | NA | 0.9 (3.6; n=1) | NA | 79 | 1.4 (n=12) |
| *V. tinus* | VL | MSA, 11/1/18, Cambridge, UK, S.N. | blue | 48 (n=2) | 14 (26.8; n=2) | 0.7 (1.3; n=1) | 1.9 (3.6; n=2) | 36 (68) | 51 | 1.08 (n=20) |
| *V. opulus* | YU | MAS 503 | red | 88 (n=3) | 0.5 (3.9; n=3) | 0.5 (4.3; n=3) | 1.0 (8.9; n=3) | 10 (83) | 88 | 3.17 (n=20) |
| *V. utile* | UCBG | 80.1247 | black (seq) | 68 (n=3) | 1.3 (4.0; n=3) | 1.4 (4.4; n=3) | 0.8 (2.7; n=3) | 28 (89) | 78 | 2.54 (n=20) |

**Table S2.** Step cost matrix implemented in Mesquite to represent the paedomorphy model. Each number indicates the relative cost of a transition between each state. *syn* = synchronously fruiting, *seq* = sequentially fruiting. Each row or column name represents a single fruit type used in the analyses; we did not model developmental type and color independently.

|  | **black (syn)** | **blue (syn)** | **black (seq)** | **red (syn)** | **yellow** |
| --- | --- | --- | --- | --- | --- |
| **black (syn)** | 0 | 1 | 2 | 2 | 3 |
| **blue (syn)** | 1 | 0 | 3 | 3 | 3 |
| **black (seq)** | 2 | 3 | 0 | 2 | 2 |
| **red (syn)** | 2 | 3 | 2 | 0 | 2 |
| **yellow** | 3 | 3 | 2 | 2 | 0 |

**Table S3.** Transition rate matrices used in the ancestral state reconstruction by maximum likelihood. *syn* = synchronously fruiting, *seq* = sequentially fruiting. Each row or column name represents a single fruit type used in the analyses; we did not model developmental type and color independently.

|  | **black (syn)** | **blue (syn)** | **black (seq)** | **red (syn)** | **yellow** |
| --- | --- | --- | --- | --- | --- |
| **black (syn)** | NA | 1 | 1 | 1 | 0 |
| **blue (syn)** | 1 | NA | 0 | 0 | 0 |
| **black (seq)** | 1 | 0 | NA | 1 | 1 |
| **red (syn)** | 1 | 0 | 1 | NA | 1 |
| **yellow** | 0 | 0 | 1 | 1 | NA |

**Table S4.** Transition rates between each fruit color state as estimated by make.simmap in *phytools* using a symmetrical model of evolution. The highest transition rates occur between red to black-synchronous, as a result of several recent origins of black-synchronous fruits from red-fruited ancestors in the Succotinus clade. Black-synchronous fruits have non-zero transition rates to every color category except yellow, which is likely due to the ancestor of Nectarotinus being reconstructed as a black-synchronous fruit. Similarly, black-sequential fruits have non-zero transition rates to every color category except blue; this makes sense in that blue fruits likely evolved from black-synchronous ancestors. We note that the ancestor to Nectarotinus may have been black-synchronous, but it may also have exhibited non-analog characteristics (such as a moderate-length immature red phase) which could have served as the developmental basis for the evolution of black-synchronous fruits to red.

|  | **black-synchronous** | **blue** | **black-sequential** | **red** | **yellow** |
| --- | --- | --- | --- | --- | --- |
| **black-synchronous** | -0.011 | 0.003 | 0.0007 | 0.007 | 0 |
| **blue** | 0.003 | -0.003 | 0 | 0 | 0 |
| **black-sequential** | 0.0007 | 0 | -0.002 | 0.0005 | 0.001 |
| **red** | 0.007 | 0 | 0.0005 | -0.008 | 0 |
| **yellow** | 0 | 0 | 0.001 | 0 | -0.001 |

**Fig. S1.** Species' colors plotted in tetrahedral color space according to a visual model of a UV-sensitive bird. Points indicate individual species plotted according to their stimulation of ultraviolet (purple corner), short-wave (blue corner), medium-wave (green corner), and long-wave (red corner) cones in the bird visual model. The colors of each point are an approximation of the RGB color value that would result from the spectrum of each species.

**Figure S2**. Volume overlap between each fruit color category in tetrahedral color space. Each panel represents a different pair of colors: a) black-synchronous and red, b) black-synchronous and blue, c) black-synchronous and black-sequential, d) red and blue, e) blue and black-sequential, and 3) black-seqential and red. In each case, the region of tetrahedral color space occupied by each fruit color category is shown; gray shaded areas represent regions of overlap. The only pair of colors that overlap in the region of color space occupied are black-sequential and black-synchronous (shown in c). Colors are as follows: purple = black-sequential, black = black-synchronous, blue = blue, red = red.


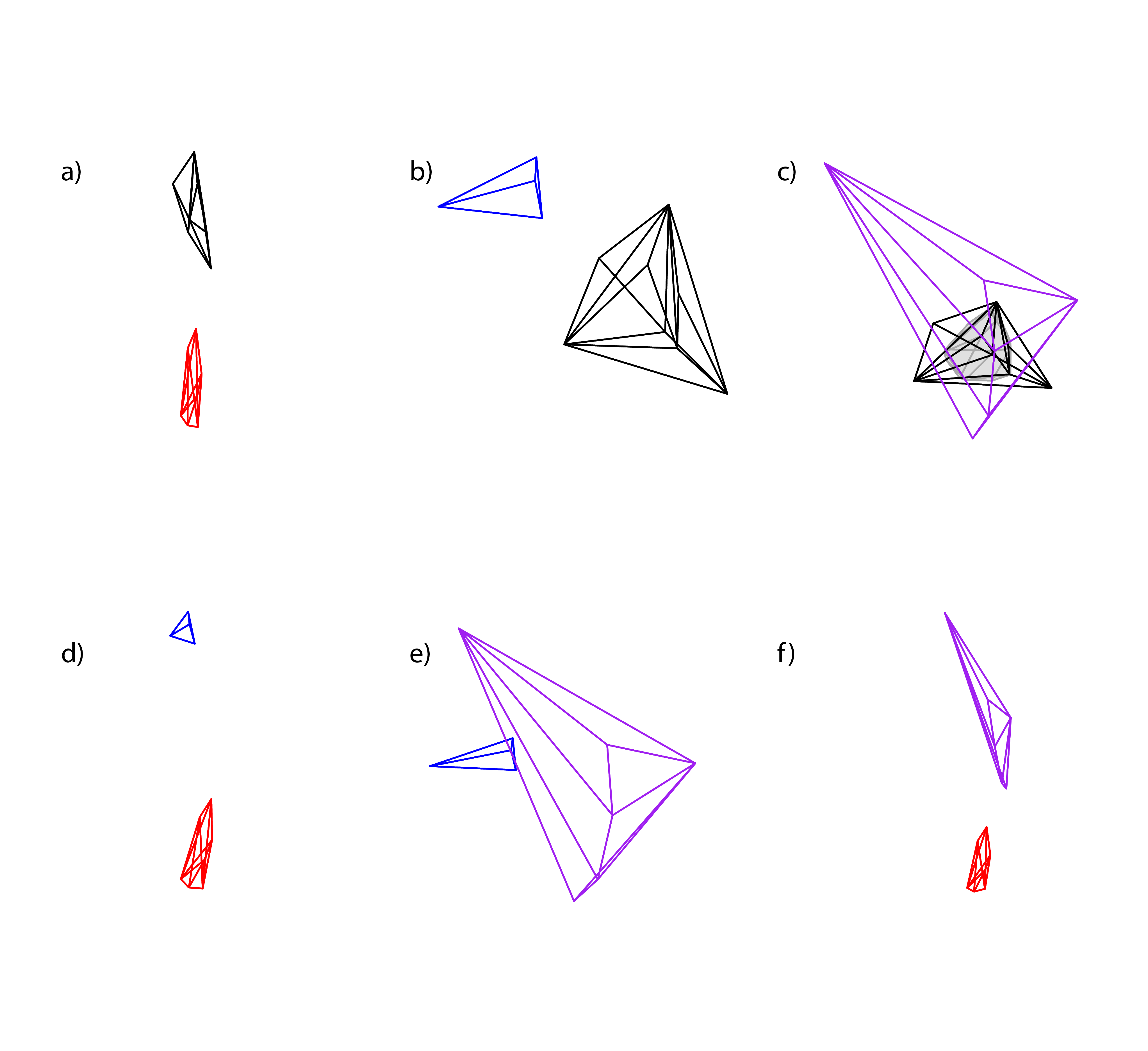

Supplement: Supplementary file 1 — Additional file 1: Table S1. Nutritional, morphological and (categorical) color data for Viburnum species, as well as accession numbers and sample sizes for each measurement. Table S2. Step cost matrix implemented in Mesquite to represent the paedomorphy model. Table S3. Transition rate matrices used in the ancestral state reconstruction by maximum likelihood. Table S4. Transition rates between each fruit color state as estimated by make.simmap in phytools using a symmetrical model of evolution.. Figure. S1. Species’ colors plotted in tetrahedral color space according to a visual model of a UV-sensitive bird. Figure S2. Volume overlap between each fruit color category in tetrahedral color space. [file 12862_2019_1546_MOESM1_ESM.docx]
